# Malakoplakie pseudotumorale du sein

**DOI:** 10.11604/pamj.2015.21.87.3966

**Published:** 2015-06-03

**Authors:** Abderrahim Elktaibi, Mohamed Reda Elochi, Mohamed Sinaa, Mohamed Allaoui, Yassir Alami, Mariam Amrani

**Affiliations:** 1 Service d'Anatomie et Cytologie Pathologiques, Institut National d'Oncologie, Rabat, Maroc; 2 Service de Chirurgie, Institut National d'Oncologie, Rabat, Maroc

**Keywords:** Malakoplakie, Michaelis-Gutmann, sein, Malakoplakia, Michaelis-Gutmann, breast

## Abstract

La malakoplakie est une maladie inflammatoire granulomateuse chronique, qui affecte généralement le tractus génito-urinaire et exceptionnellement la glande mammaire. Il faut savoir évoquer ce diagnostic devant une mastite pseudotumorale. Sa définition est anatomopathologique. Nous rapportons un cas inhabituel de malakoplakie mammaire chez une patiente ayant des antécédents de tuberculose. L'imagerie était en faveur d'une mastite carcinomateuse. L'analyse histologique de la biopsie mammaire révélait une inflammation granulomateuse faite d'histiocytes renfermant des granulations pathognomoniques de Michaelis-Gutmann. La patiente était mise sous traitement médical à base de ciprofloxacine avec bonne évolution clinique et radiologique après un recul d'un mois.

## Introduction

La Malakoplakie ou encore appelée malakoplasie [1] est une maladie inflammatoire granulomateuse chronique peu commune, qui affecte généralement le tractus génito-urinaire (environ 75 % des cas rapportés) et moins fréquemment les autres sites tels que le tractus gastro- intestinal, la thyroïde, le pancréas, le foie, le cerveau, les ganglions lymphatiques, les surrénales, la peau, les os, le rétropéritoine et le sein. Elle représente un déficit dans la digestion macrophagique des bactéries mais sa pathogénie précise n'est pas encore claire [2]. La définition de la malakoplakie est anatomo pathologique [3] caractérisée par la présence de cellules de Von Hansemann renfermant des corps de Michaelis et Gutmann. Contrairement à cette spécificité anatomo- pathologique, la malakoplakie n'a rien de spécifique sur le plan clinique ou radiologique. Le traitement repose essentiellement sur l'antibiothérapie en l'absence de destruction importante de l'organe atteint [4]. A travers un cas exceptionnel de localisation mammaire pseudotumorale isolée, nous discutons les aspects physiopathologiques, diagnostiques et thérapeutiques de cette affection.

## Patient et observation

**Figure 1:**
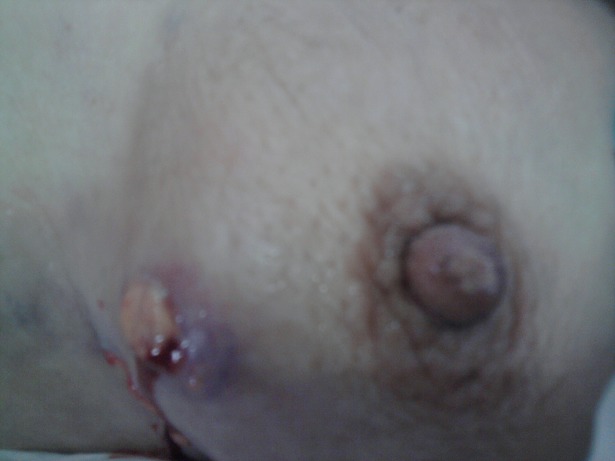
Photographie du sein gauche montrant un aspect en peau d'orange avec fistulisation à la peau

**Figure 2:**
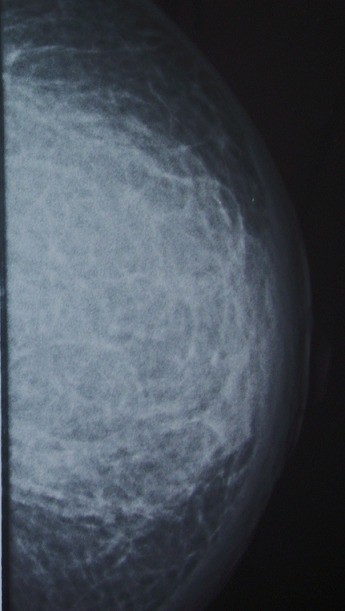
Mammographie montrant une augmentation de la densité du sein avec infiltration de la graisse sous cutanée évoquant une mastite tumorale

**Figure 3:**
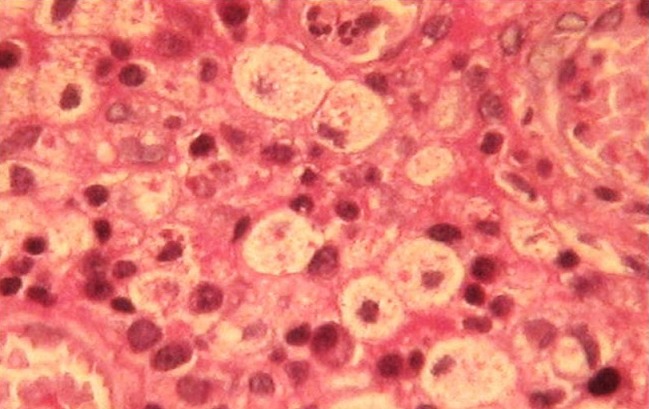
Microphotographie au fort grossissement montrant des histiocytes renfermant les corps de Michaelis-Gutmann positifs à la coloration de PAS (periodic acid schiff)

**Figure 4:**
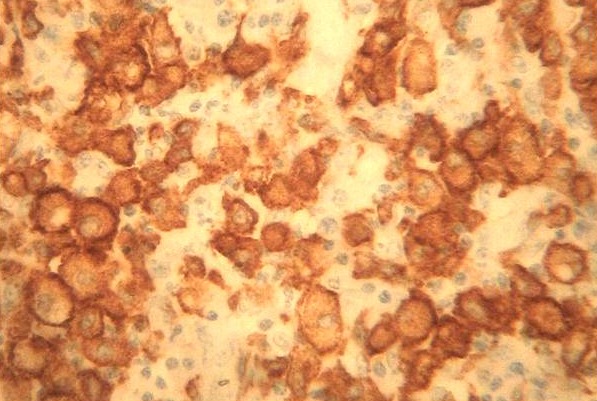
Immunomarquage positif des cellules histiocytaires par l'anticorps anti CD163

Madame BK, âgée de 52 ans; ayant un antécédent de pneumonectomie gauche pour aspergillose pulmonaire sur séquelle tuberculeuse en novembre 2008. Elle présentait 4 mois auparavant une tuméfaction douloureuse du sein gauche. L'examen clinique montrait un sein augmenté de volume, douloureux à la palpation, chaud et inflammé en peau d'orange avec fistulisation à la peau et adénopathie axillaire homolatérale (Figure 1). La mammographie objectivait une augmentation de la densité mammaire, épaississement cutané et infiltration de la graisse sous cutanée évoquant une mastite carcinomateuse (Figure 2). Le complément échographique mettait en évidence un processus lésionnel mal limité hypoéchogène homogène prenant tout le sein gauche, associé à un aspect echogène de la graisse adjacente et à un épaississement sous cutané avec adénomégalie axillaire gauche. L'aspect mammo-echographique était en faveur d'une mastite d'allure tumorale. Une biopsie a été réalisée et l'examen anatomopathologique montrait une réaction inflammatoire faite d'une nappe d'histiocytes spumeux (cellules de Von Hansemann) aux noyaux réguliers faiblement nucléolés, au cytoplasme microvacuolaire renfermant des concrétions éosinophiles PAS (periodic acid schiff) positif (corps de Michaelis et Gutmann) (Figure 3). A l'étude immunohistochimique, ces cellules expriment l'anticorps anti CD163; marqueur des cellules histiocytaires (Figure 4). L'aspect morphologique était en faveur d'une malakoplakie. La patiente a été mise sous fluoroquinolones (ciprofloxacine 500mg ×2/j) avec bonne évolution clinique et radiologique après un recul d'un mois.

## Discussion

Décrite pour la première fois en 1902 par Michaelis et Gutmann. La malakoplakie dans sa localisation extra-urinaire n'a été décrite qu'à partir de 1985 [5]. Cette maladie s'observe à tout âge avec un pic de fréquence à la cinquième décade [6]. Toutes localisations confondues, les deux sexes sont également atteints. Les lésions extra-urinaires sont moins fréquentes et s'observent dans 40 % des cas. Elles intéressent le tube digestif dans 20 % des cas et le rétropéritoine dans 10 % des cas [7]. Des cas isolés ont été rapportés au niveau de la peau, la vulve, le vagin, l'endomètre, la surrénale et le cerveau [4]. La localisation mammaire est exceptionnelle. Notre observation représente le 4ème cas de la malakoplakie mammaire selon les données de la littérature; 2 observations italiennes montraient des lésions évocatrices de la malakoplakie au cours de l'évolution d'une mastite puerpérale [8]. Le 3ème cas a été rapporté par Faucher et al. en 1993 [9]. Sur le plan physiopathologique, la malakoplakie est due à un déficit de la fonction macrophagique [3]. En effet, La bactérie est phagocytée par le macrophage et se retrouve dans une vacuole cytoplasmique (phagosome). La fusion phagosome et lysosome semble se faire mais la "digestion" des éléments bactériens ne peut parvenir à son terme, ce qui aboutit à l´accumulation d´inclusions d´abord simples puis qui s´incrustent de fer et de calcium donnant les corps de Michaelis-Gutmann. Des facteurs favorisants comme une maladie intercurrente ou une immunosuppression ont été rapportées dans 40% des cas [6]. DORÉ dans sa série rapportait des antécédents de diabète, d'éthylisme, de cancer, de corticothérapie et de tuberculose, comme c'est le cas dans notre observation. Des cas récents ont été rapportés chez des transplantés cardiaques, rénaux et chez des patients atteints de SIDA [3]. La malakoplakie n'offre sur le plan clinique aucune spécificité [4]. Elle est fonction du siège des lésions. L'atteinte mammaire isolée est exceptionnelle et se présente généralement comme une mastite diffuse avec aspect en peau d'orange ou lésion abcédée. Son diagnostic différentiel se pose essentiellement avec une mastite carcinomateuse ou tuberculeuse. Histologiquement, le granulome malakoplasique est caractérisé par la présence des cellules de Van Hansemann [4]. Ces cellules sont des histiocytes à large cytoplasme, riche en granulations éosinophiles et des enclaves basophiles de grande taille contenant les corps de Michaelis et Gutmann (CMG). Ces derniers donnent aux histiocytes l'aspect en cible ou en un œil d'oiseau et constituent la lésion pathognomonique de la malakoplakie [7]. L'étude de ces CMG a montré qu'ils sont faits de débris bactériens ou de germes entiers qui peuvent être source de récidive à l'arrêt du traitement antibiotique [4]. L'évolution peut se faire vers la guérison lorsque l´infection est correctement traitée (antibiotiques adaptés, traitement suffisamment long). Lorsque l´infection persiste, ou lorsque les troubles immunologiques sont marqués, l'évolution peut se faire vers l'aggravation et la destruction de l'organe atteint [5]. En dehors des complications, le traitement médical est actuellement proposé en première intention, il est basé sur l'utilisation des antibiotiques adaptés aux germes responsables: le plus souvent il s'agit des fluoroquinolones (1g/j) ou du cotrimoxazole triméthoprime. L'utilisation du bethanéchol choride a été peu étudiée ainsi que sa réelle efficacité in vivo; son rôle serait d'améliorer la fonction macrophagique par une augmentation du GMPc; ce dernier stimule la synthèse du tumor necrosis factor (TNF) qui favorise l'activité bactéricide des macrophages [3]. L'adjonction de l'acide ascorbique à ces médicaments, semble pour certains améliorer la performance du traitement. Le traitement chirurgical quant à lui, est indiqué en cas de destruction de l'organe atteint [4].

## Conclusion

La malakoplakie est une pathologie rare, encore dans sa la localisation mammaire. Contrairement à sa spécificité histologique, elle ne présente aucun tableau clinique ou radiologique univoque. Le polymorphisme clinique et le terrain immunodéprimé, doivent attirer l'attention et permettre au pathologiste d'évoquer ce diagnostic. Le traitement de la malakoplakie repose essentiellement sur une antibiothérapie associée aux cholinergiques. L'exérèse chirurgicale n'est réalisée qu'en cas de complications ou de destruction de l'organe atteint.
